# Short-term and delayed effects of mother death on calf mortality in Asian elephants

**DOI:** 10.1093/beheco/arv136

**Published:** 2015-08-20

**Authors:** Mirkka Lahdenperä, Khyne U. Mar, Virpi Lummaa

**Affiliations:** ^a^Section of Ecology, Department of Biology, University of Turku, Turku, Finland and; ^b^Department of Animal and Plant Sciences, University of Sheffield, Alfred Denny Building, Western Bank, Sheffield S10 2TN, UK

**Keywords:** ageing, fertility, parental care, reproductive cessation, senescence.

## Abstract

Long-lived, highly social species with prolonged offspring dependency can show long postreproductive periods. The Mother hypothesis proposes that a need for extended maternal care of offspring together with increased maternal mortality risk associated with old age select for such postreproductive survival, but tests in species with long postreproductive periods, other than humans and marine mammals, are lacking. Here, we investigate the Mother hypothesis with longitudinal data on Asian elephants from timber camps of Myanmar 1) to determine the costs of reproduction on female age-specific mortality risk within 1 year after calving and 2) to quantify the effects of mother loss on calf survival across development. We found that older females did not show an increased immediate mortality risk after calving. Calves had a 10-fold higher mortality risk in their first year if they lost their mother, but this decreased with age to only a 1.1-fold higher risk in the fifth year. We also detected delayed effects of maternal death: calves losing their mother during early ages still suffered from increased mortality risk at ages 3–4 and during adolescence but such effects were weaker in magnitude. Consequently, the Mother hypothesis could account for the first 5 years of postreproductive survival, but there were no costs of continued reproduction on the immediate maternal mortality risk. However, the observed postreproductive lifespan of females surviving to old age commonly exceeds 5 years in Asian elephants, and further studies are thus needed to determine selection for (postreproductive) lifespan in elephants and other comparably long-lived species.

## Lay Summary

Like humans, elephants are long lived, invest heavily in offspring, and often survive well past last birth, but why do postreproductive lifespans evolve? We show that the proposed higher costs of reproduction to survival of old mothers and need for long parental care of offspring are insufficient to explain the full length of postreproductive lifespan in Asian elephants. Further studies are needed to quantify the evolutionary pressures on postreproductive survival in elephants and other long-lived species.

## INTRODUCTION

Highly social, long-lived mammals are characterized by extended parental care and investment (Carey 2003). In these species, a considerable mismatch between reproductive and somatic senescence can evolve (Lee 2003; Bourke 2007). Accordingly, some of the longest-lived mammals—killer whales (*Orcinus orca*), short-finned pilot whales (*Globicephala macrorhyncus*), and humans—are known to exhibit menopause (Marsh and Kasuzya 1986; Ward et al. 2009). Several adaptive hypotheses have been proposed to explain the evolution of such a life history in these species (e.g., the Mother [Williams 1957; Packer et al. 1998; Moss de Oliveira et al. 1999; Peccei 2001] and Grandmother hypotheses [Hawkes et al. 1998; Foster et al. 2012] and the Reproductive Conflict hypothesis [Cant and Johnstone 2008; Lahdenperä et al. 2012]). However, other equally long-lived, social species such as the 2 species of elephants do not show a similar kind of abrupt loss of total fecundity at old age (Moss 2001; Lahdenperä et al. 2014). Nevertheless, although the postreproductive lifespan can be relatively long compared with many other mammals also in elephants (Cohen 2004; Lahdenperä et al. 2014), the adaptive hypotheses for the evolution of postreproductive lifespan have not been tested in long-lived terrestrial species other than humans (see Ward et al. 2009; Foster et al. 2012 for tests in killer whales).

The Mother hypothesis was originally proposed by Williams (1957) as an explanation for the evolution of menopause in humans, but it also predicts that extended maternal care can select for longer female postreproductive lifespan. Accordingly, when costs of reproduction increase with age and offspring fitness is linked to their amount of maternal care, continued survival well beyond last reproductive attempt can be selected for over time. In other words, selection should maintain somatic functions long enough after reproductive cessation to rear those offspring that are still dependent on the mother. Consequently, postreproductive lifespan should be the greatest in species with most prolonged infant dependency. Support for this theory is patchy, however. In humans, with the longest postreproductive lifespan of >25 years (e.g., Lahdenperä et al. 2014), the maternal effects alone, including higher risk of dying from childbirth with age and the offspring need for maternal care, are estimated to be too weak to select for the evolution of menopause and the subsequent decades long postreproductive lifespan (Lahdenperä et al. 2011). For a few other species such as olive baboons (*Papio cynocephalus anubis*) and African lions (*Panthera leo*), some evidence suggests that their life expectancy after the decrease in reproductive capacity could match with the length of infant dependency (Packer et al. 1998). However, this hypothesis, as an explanation for postreproductive lifespan, has not been tested in highly social and long-lived terrestrial species with the maximum lifespan approaching human longevity.

Asian elephants (*Elephas maximus*) have a long lifespan (maximum of 80 years), generally live in social groups consisting of related females and calves (de Silva et al. 2011) and have long offspring dependency (Sukumar 2003a). The calves depend on their mothers and other family members for social support, survival, and learning for the first years of life. Female calves remain in their families (not always when they grow up, de Silva et al. 2011), whereas males disperse during adolescence, approximately at age 10 (Mellen and Keele 1994) and play no role in rearing offspring. Fecundity clearly decreases in old Asian elephant females (Robinson et al. 2012; Hayward et al. 2014; Lahdenperä et al. 2014), but some of the females are still capable of reproducing after reaching 60 years (Lahdenperä et al. 2014). Nevertheless, postreproductive lifespan can be relatively long, at the population level 17 years after age of 47, when 95% of population fecundity has been realized and at the individual level 11 years for those females living beyond 40 years (Lahdenperä et al. 2014). In line with this, according to another measure, postreproductive representation (PrR; Levitis and Lackey 2011), which is suitable for comparing postreproductive survival in species with different longevities, 13% (PrR 0.13) of adult Asian elephant females, which had reproduced, in a semicaptive population in Myanmar at any time point were postreproductive, and thus, one-eighth of adult female lifespan could be spent as postreproductive (Lahdenperä et al. 2014). Although this is clearly lower than in species with known menopause and decades long postreproductive lifespan such as humans (PrR between 0.3 and 0.78 depending on the population, Levitis and Lackey 2011), killer whales (PrR 0.22, Croft et al. 2015), and short-finned pilot whales (PrR 0.28, Levitis and Lackey 2011), this is still longer than in many primates living in wild or semiwild conditions, such as chimpanzees (*Pan troglodytes*) often claimed to exhibit menopause (e.g., Videan et al. 2006) (PrR 0.018, Levitis and Lackey 2011). However, it is unknown whether the period of postreproductive lifespan corresponds to the length of time needed to ensure offspring survival in elephants along the lines of the Mother hypothesis predictions, and similarly what the consequences of maternal death for offspring at varying ages are.

In this study, we test the Mother hypothesis with a multigenerational longitudinal data on Asian elephants from timber camps of Myanmar. First, we determine the costs of reproduction with female age by investigating the age-specific risk of dying from calving-related causes within 1 year from birth for females. Second, we investigate the effects of mother loss on calf survival during dependency. We investigate these effects by measuring the short-term and delayed effects of mother loss for calf survival. Short-term mortality effects were measured as the probability that a calf would die during current or the subsequent year following maternal death until age 5. Delayed effects were measured in 2 ways, first, as the probability that an offspring would die during the following years, until age 6 years, given they had already survived the same and the subsequent year following maternal death. Finally, delayed effects of maternal loss during the most susceptible time, 0–3 years, were investigated on the following survival between ages 6 and 9 in adolescent elephants. We investigate these to evaluate whether in long-lived Asian elephants elderly females suffer increased mortality costs of reproduction; if extended maternal care could have selected for longer female postreproductive life span; and what are the outcomes for individual survival of early mother loss.

## METHODS

### Study population

We utilized a large multigenerational demographic dataset on a semicaptive Asian elephant population used in timber industry in Myanmar (formerly Burma) (Mar et al. 2012; Robinson et al. 2012; Mumby et al. 2013a, 2013b; Hayward et al. 2014; Lahdenperä et al. 2014). Myanmar has the largest captive Asian elephant population in the world and the second largest total population after India. The dataset has been collected by using the elephant logbooks and annual extraction reports archived and maintained by the Extraction Department, Myanma Timber Enterprise, and covers the life history of succeeding generations of captive timber elephants born to the population or captured from the wild for over a century. The dataset contains information on each individual, in each generation of: registration number and name of elephant; origin (captive born [CB; 52%] or wild caught [WC; 48%]); date and place of birth; mother’s registration number and name; method (if WC), age, year, and place of capture; year or age of taming; dates and identities of all calves born; date of death or last known date alive and cause of death. Although the ages of CB elephants are known because precise dates of birth are recorded, elephants caught from the wild are aged by comparing their height and body condition at the time of capture with captive elephants of known age.

The timber elephants of Myanmar live in forest camps, where they are used during the day as riding, transport, and draft animals. At night, the elephants forage in forests in their family groups unsupervised where they find food and encounter tame and wild conspecifics. Their age-specific survival rates are close to those reported for wild elephant populations (Clubb et al. 2008) and the causes of death include natural causes such as starvation, tiger attacks, and a range of recorded diseases, as well as accidents (for death causes of calves see Mar et al. 2012; for seasonal variation in all age groups see Mumby et al. 2013a). Breeding rates are natural with humans not intervening or helping with matings, and most calves are thought to be sired by wild bulls. Calves born in captivity are cared for by their biological and allomothers, and suckle until lactation no longer supports their demands. Working females are given rest from midpregnancy (11 months into gestation) until the calves reach their first birthday (Toke Gale 1971). Mothers are then used for light duties but allowed to nurse the calves on demand; neither mothers nor calves have traditionally been supplemented with food by their human caretakers. Although it is possible that female longevity and orphan survival are influenced by the animal husbandry practices that the population is subject to, the fact that both maternal and calf mortality rates generally resemble those reported for wild elephant populations (Clubb et al. 2008) suggests that the maternal work effort is unlikely to have substantial effect on the mother’s capability to take care of the calf. The work effort is more likely to affect the fertility rates than the mortality rates, which is confirmed by a finding that whereas body condition and mortality patterns in the population follow seasonal changes in rainfall and food availability, conception rate is increased during the annual rest period (Mumby et al. 2013b). Importantly, however, workload may only reduce reproductive rate of prime aged, but not old, females, given females enter workforce at 17 years and all females 55 years and older are retired from work.

Mahouts or human caretakers neither intervene nor assist in the calving/nursing processes. The calves are generally weaned at the age of 4 or earlier when they are capable of independent foraging. They are separated from the mother and tamed between ages 4 and 5 and then given a mahout, name, a log-book, and registration number and trained and used for light work as baggage elephants until age 17. Unfortunately, the exact dates of weaning/training are currently not included in the data, and thus, we are unable to separate their effects on calf mortality. Although the elephants normally forage independently in the forest and are not nutritionally provisioned by their caretakers, if the mother dies early, the caretakers try to encourage allomothers to take care of the calf. This may contribute to somewhat higher observed orphan survival compared with wild African elephants (*Loxodonta africana*) (Lee and Moss 2011); however, comparable data on Asian elephants are not available to confirm the differences. By age 17, the elephants are put into workforce as full-working elephants. The elephants are retired at age 55 (Toke Gale 1971; Mar 2007) and live out the rest of their lives in comparative freedom, but their logbooks are maintained until death.

We analyzed 1872 calves (F = 936, M = 936) born between 1947 and 2011 to 857 mothers and excluded stillborn, calves born to mothers captured before 1952 (with only limited records available prior to this year) and calves with mistakes or occasional missing information on sex or own or mother’s exact or censored lifespan after calf’s birth. These calves come from 39 timber extraction areas within 10 regions (out of a total of 14) in Myanmar: Ayeyarwa, Bago, Chin, Kachin, Magway, Mandalay, Rakhine, Sagaing, Shan, Tanin, and a few with unknown region. In the analyses, Chin, Rakhine, Tanin, and Ayeyarwa were all grouped together because of low sample size and their location on coastal regions. The youngest first-time mother in the sample was 5.3 years although reproduction before age 10 was rare, similarly to data from other elephant populations (Moss 2001; Sukumar 2003b). The oldest reproducing female was 64.9 years, and the mean maternal age of all calves was 28.5±9.5 years; median: 27.0 years. The average interbirth interval was 5.92±3.11 years (median = 5.26), and the maximum number of calves for the same mother was 9.

### Statistical analyses

All statistical analyses were conducted using SAS (SAS Institute Inc., release 9.3, 2002–2010). Fixed terms were retained in models only if they improved explanatory power, determined using Akaike information criteria (discrete time survival analyses). Interactions were only tested if they were specifically predicted by the Mother hypothesis and expected based on previous studies in Asian elephants.

First, we quantified the age-specific mortality and fertility to illustrate the overall trends in the population. We included all calves and their mothers for quantification of population-level mortality (*n* = 2729) and all mothers for age-specific fertility (*n* = 857). The ages above 70 were grouped together in both analyses because of small sample size (*n* = 8 individuals). For mortality function, we included those individuals still alive at the end of the study and those disappearing before death as censored observations (right censoring) and captured individuals entering the population at the age of capture (left censoring), whereas CB individuals entered the analysis at birth. We calculated the age-specific fertility as the total number of (live-born) calves born during each year divided by the number of mothers alive at the end of each year. For age-specific fertility function, we included mothers captured from the wild the first time at their capture age and thus the total sample size was adjusted to the actual number of mothers in a population at each age.

### Age-specific risk of maternal death after calving

In line with the predictions of the Mother hypothesis, we determined the age-specific rate of calving-related maternal mortality in Asian elephants. Elephants have the longest gestation period among terrestrial mammals averaging 22 months (Lueders et al. 2012). Abnormal deliveries and stillbirths are major issues in captive elephant populations (Hermes et al. 2008), and thought to arise from the unique anatomical features of elephants and the risk factors associated with advancing age of primiparous females in captivity. A survey data suggest that animals that reproduced first time after age 30 experienced higher incidences of stillbirths and abnormal deliveries (Doyle et al. 1999). Abnormal labor (dystocia) often leads to fetal and maternal death (Hermes et al. 2008). Our analysis of the age-specific risk of dying from calving was conducted using 2426 births by 1038 mothers. A mother was defined as having died in calving or calving-related conditions if she died within 1 year following parturition (35 mothers, see also Packer et al. 1998 for the same definition in baboons and lions). The time period was extended to 1 year because there were only 10 mothers dying within 6 weeks (definition used for birth-related deaths in humans; Knodel 2002; Lahdenperä et al. 2011) from birth of their calf.

The effect of maternal age on the probability of dying in calving-related causes was investigated using a generalized linear mixed effects model (GLMM) with a pseudo-likelihood estimation technique in which the data were fitted to a binomial error structure with logit link function (with GLIMMIX procedure in SAS). Linear and quadratic functions of female age were fitted as the main fixed terms of interest, although birth order, birth sex, birth year (cohort), previous birth interval, female’s origin (CB vs. WC), age at first reproduction (AFR), and working area (state) were fitted as potential controlled terms. Previous birth interval was categorized as short, medium, long, and firstborn categories based on 25% and 75% quartiles of birth-interval length (3.85 and 7.30 years, respectively). Female age and birth order were fitted as continuous variables, but in the case of the former, deliveries over the age of 60 were pooled, and in the case of the latter, birth orders after the fifth delivery were pooled. In both cases, this was due to low numbers of births occurring at such extremes. Maternal identity was included as a random term to account for repeated births by the same female.

### Consequences of maternal death for offspring

The second prediction of the Mother hypothesis, the effect of mother loss on calf mortality at different ages, was investigated in 2 ways. First, we examined the effects of mother loss on the immediate short-term mortality of calves from birth until age of 5 years. Short-term mortality effects were defined as the probability that a calf would die during current or the subsequent year following maternal death. Second, we examined whether mother loss had delayed effects on calf mortality before age 6 (3–5). Delayed effects were defined as the probability that a calf would die during the following years, until age 6 years, given it had already survived the same and the subsequent year following maternal death. Third, we investigated whether mother loss during early ages (0–3) had delayed effects on calf mortality from age 6 until age 10 during adolescence (6–9). The annual survival of 1872 calves was known from birth to age at death or censoring point along with information on their mother’s survival. In all analyses, we used discrete time survival data and performed the analyses with maximum likelihood estimation technique in GLMM (GLIMMIX procedure of SAS) with binomial error structure and logit link function to determine the effect of mother loss on the mortality of the calves.

#### Short-term effects of mother loss

The effect of mother loss on short-term offspring mortality (i.e., within the same or following year) was investigated to find out whether there are immediate survival costs for offspring. First, we used discrete time survival analysis (also known as event history analysis), which allows a sensitive analysis of the effects of time-dependent variables, such as the presence of relatives (see below), on the calf’s probability of dying over discrete time intervals, while accounting for repeated sampling of different offspring born to the same mother (hence experiencing a common environment, Singer and Willett 2003). This analysis allowed us to estimate the calf’s risks of dying in each year from birth to age of 5 years in the presence versus absence of a living mother. For each year from birth to 5 years (5 time intervals for each offspring), the survival of each calf was coded as survived versus died during the observation year (1/0) or missing (when the death had already occurred or because of censoring). Each year illustrates the probability of the calf dying within the following full year (year 0: 0–0.99, year 1: 1–1.99 etc.). The 0–4 analysis was carried out on 7606 data points with information on all controlled variables (*n* = 1813 calves from 838 mothers), 440 calves dying during this interval. At age 0, 19 calves lost a mother; at age 1, 21; at age 2, 30; at age 3, 26, and finally at age 4, 27 calves lost a mother.

In the analysis, an assessment of the interaction between maternal presence and calf age on calf mortality provides an indication of whether calves are more likely to die at a given age after maternal loss compared with if they lost their mother at other ages and thus whether the effect of maternal death changes with calf age. Therefore, mother presence, calf age, and their interaction were fitted as the primary fixed effects of interest. In addition, the analysis controlled for the following variables: previous birth-interval length (categorized as short [<25% quartile, 3.86 years], medium [3.86–7.40 years], long [>75% quartile, 7.40 years], and firstborn; known to be linked to calf survival [Mar et al. 2012]), calf birth cohort (cohorts: 1947–1969, 1970–1979, 1980–1989, 1990–2011), sex and birth order (birth orders after the fifth delivery were pooled), mother’s origin (CB vs. WC), mothers age at the time of birth and squared mother’s age, mother’s working area (8 areas), and a random term of mother’s identity.

#### Delayed effects of mother loss

We next investigated whether maternal death had delayed effects on calf mortality during development (up to age 6; this age was selected because calves were separated from their mothers around age 5). The primary difference here to the analysis above was that calf mortality was investigated at least 2 years following maternal death (all calves which lost a mother and were included in the analyses had survived the same and the subsequent year after mother’s death). Therefore, the mother’s presence was coded as being alive or having died at least 2 years before each observation year (e.g., at age 5, the mother had died at age 3 or before) (3 observations of mother presence for each calf from ages 3–5 years). The analysis begins from the earliest possible age, age 3, because the maternal death had to have occurred at least 2 years ago and at age 3, the mother had died at age 1 or before. All calves included in this analysis survived until age 3. The analysis was carried out on 3787 data points with information on all controlled variables (*n* = 1389 calves from 725 mothers), and 219 calves died during this interval. From all calves surviving to age 3, 18 calves at age 3 had lost a mother at least 2 years before and survived, 38 at age 4, and 60 at age 5.

Finally, the effects of maternal death during the most detrimental age-category of mother loss (0–3 [3.99]; see Short-term effects of mother loss in Results for details) were assessed on calf mortality during ages 6–9 among older juveniles and adolescents (in the wild, male calves disperse during adolescence at c. 10 years) (Mellen and Keele 1994). This analysis was conducted on 3950 data points (1085 calves from 637 mothers) from which 42 had lost a mother between ages 0 and 3 and survived to age 6. For those calves, whose mother was coded as alive, the mother had survived at least to age 4 and until the calf was weaned. In both of the delayed effect analyses, the same controlled terms were considered as for the short-term analyses outlined above.

## RESULTS

**Figure 1:**
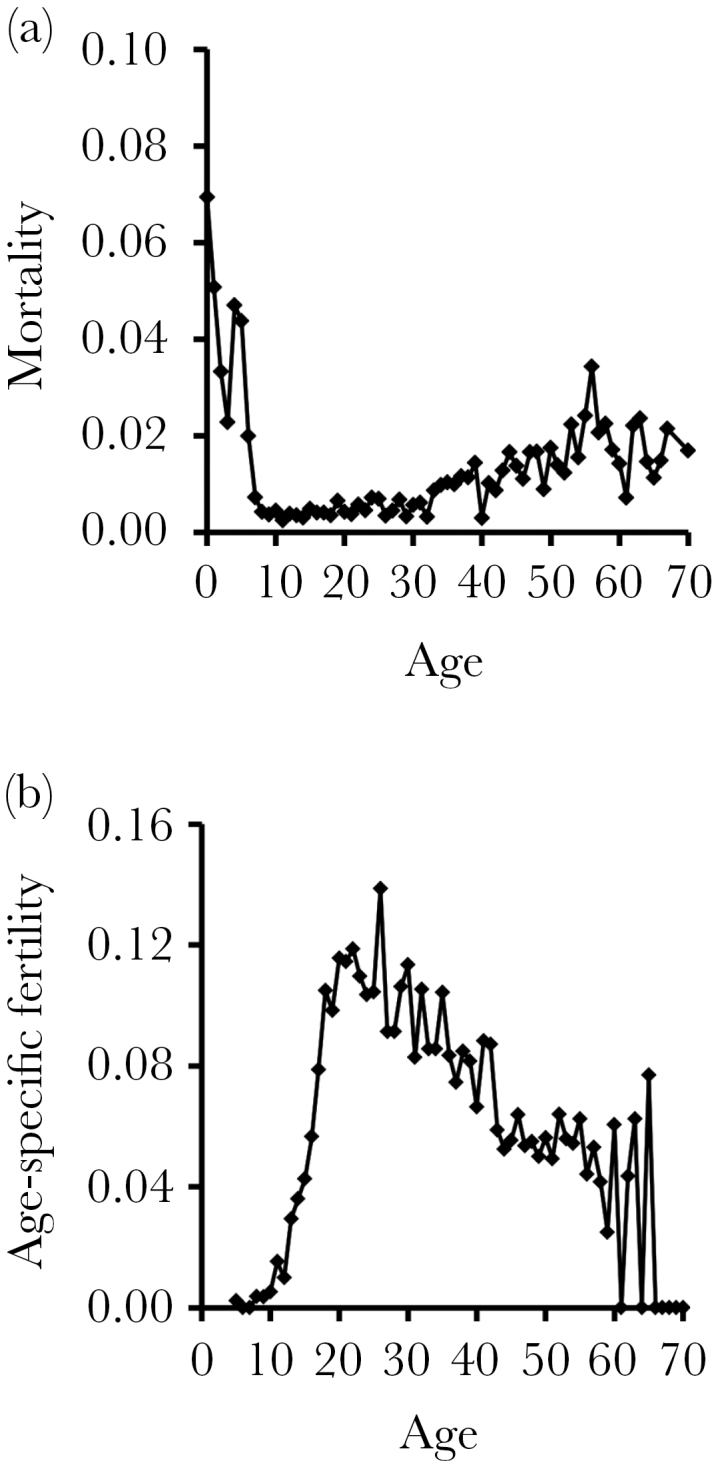
Age-specific mortality and fertility in Asian elephants. (a) Mortality of mothers and their calves by age (*n* = 2729). (b) Fertility of mothers by age (*n* = 857).

In general, the age-specific mortality of elephants was highest during the first 5 years of life, gradually increasing again from around age of 30 years onwards (Figure 1a) and following the expected pattern in mammals (Bronikowski et al. 2011). The female fertility peaked around age 20–25 years and slowly decreased until the age of latest birth in the sample, 65 years (Figure 1b).

### Age-specific risk of maternal death after calving

The death of a female within 1 year after calving was rare as only 1.4% of all 2426 births ended in maternal mortality, equating to 3.4% of the 1038 mothers dying. Moreover, only 10 of the 1038 mothers, that is 1.0%, died within 6 weeks after calving. The risk of dying within 1 year after calving varied among different birth cohorts and mother’s working area, and our analyses adjust for such variation (Table 1). The risk of dying within 1 year after calving did not depend on the age of the mother, and therefore, ageing females were not at a higher risk of dying from birth-related causes compared with younger females (Table 1).

**Table 1:** Factors affecting female’s risk of dying within 1 year from calving in semicaptive timber elephants in Myanmar (*n* = 2426 births from 1038 mothers)

Term	Estimate ± SE	Statistic (*F*_numdf,dendf_)	*P* value
Female’s age	0.0093±0.018	0.27_1,1378_	0.60
Female’s working area	Random variation	2.41_7,1378_	0.019
Birth cohort	Random variation	2.28_3,1378_	0.078
Constant	−2.78±0.82		
Female’s age^2^	0.0018±0.0011	2.53_1,1377_	0.11
Female’s origin (CB vs. WC)	−0.61±0.43	2.00_1,1378_	0.16
Female’s AFR	0.037±0.027	1.91_1,1368_	0.17
Offspring sex (female vs. male)	0.49±0.36	1.85_1,1377_	0.17
Birth order	−0.19±0.15	1.56_1,1377_	0.21
Birth order^2^	0.11±0.078	1.86_1,1376_	0.17
Previous birth interval		1.14_3,1375_	0.33

Estimates (positive reflect increasing mortality risk) are provided for variables and 2-level factors. Terms retained and rejected in the final model are shown above and below the constant, respectively. Mother’s identity was fitted as a random term. SE, standard error.

### Consequences of maternal death for offspring

#### Short-term effects of mother loss

In Asian elephants in Myanmar, 74% of calves survived to age of 5 years. Of all calves, 7% lost their mother before reaching age 5. The probability of calves dying during their first 5 years of life was influenced by maternal and calf age, calf sex, and previous birth-interval length (Table 2). The calves of older mothers and male calves had higher risk of dying (odds ratio for male calves 1.15) (Table 2; see also Mar et al. 2012).

**Table 2:** Discrete time survival model of the short-term effects of mother loss on offspring risk of death during 0–4 (4.99) years in semicaptive timber elephants in Myanmar (total *n* = 7606 observations [1813 calves and 838 mothers])

Term	Estimate ± SE	Statistic (*F*_numdf,dendf_)	*P* value
Mother’s death	2.71±0.50	35.59_1,6755_	<0.0001
Calf age	−1.07±0.14	54.14_1,6755_	<0.0001
Calf age^2^	0.17±0.030	33.14_1,6755_	<0.0001
Mother’s age	0.015±0.0058	6.38_1,6755_	0.012
Previous birth interval		3.90_3,6755_	0.0085
Previous birth interval × calf age		6.51_3,6755_	0.0002
Sex (female vs. male)	−0.14±0.10	4.33_1,6755_	0.037
Mother’s death × calf age	−0.57±0.18, Figure 2a	10.36_1,6755_	0.0013
Mother’s death × calf sex	Figure 2b	2.25_1,6755_	0.13
Constant	−2.35±0.24		
Mother’s origin (CB vs. WC)	−0.14±0.11	1.78_1,6755_	0.18
Birth cohort	Random variation	1.52_3,6752_	0.21
Birth order	−0.049±0.057	0.73_1,6754_	0.39
Mother’s age^2^	−0.000033±0.00042	0.01_1,6754_	0.94
Mother’s working area	Random variation	0.46_7,6754_	0.86

Estimates (positive reflect increasing mortality risk) are provided for variables and 2-level factors. Terms retained and rejected in the final model (determined using AIC) are shown above and below the constant, respectively. Mother’s identity was fitted as a random term. AIC, Akaike information criteria; SE, standard error.

**Figure 2:**
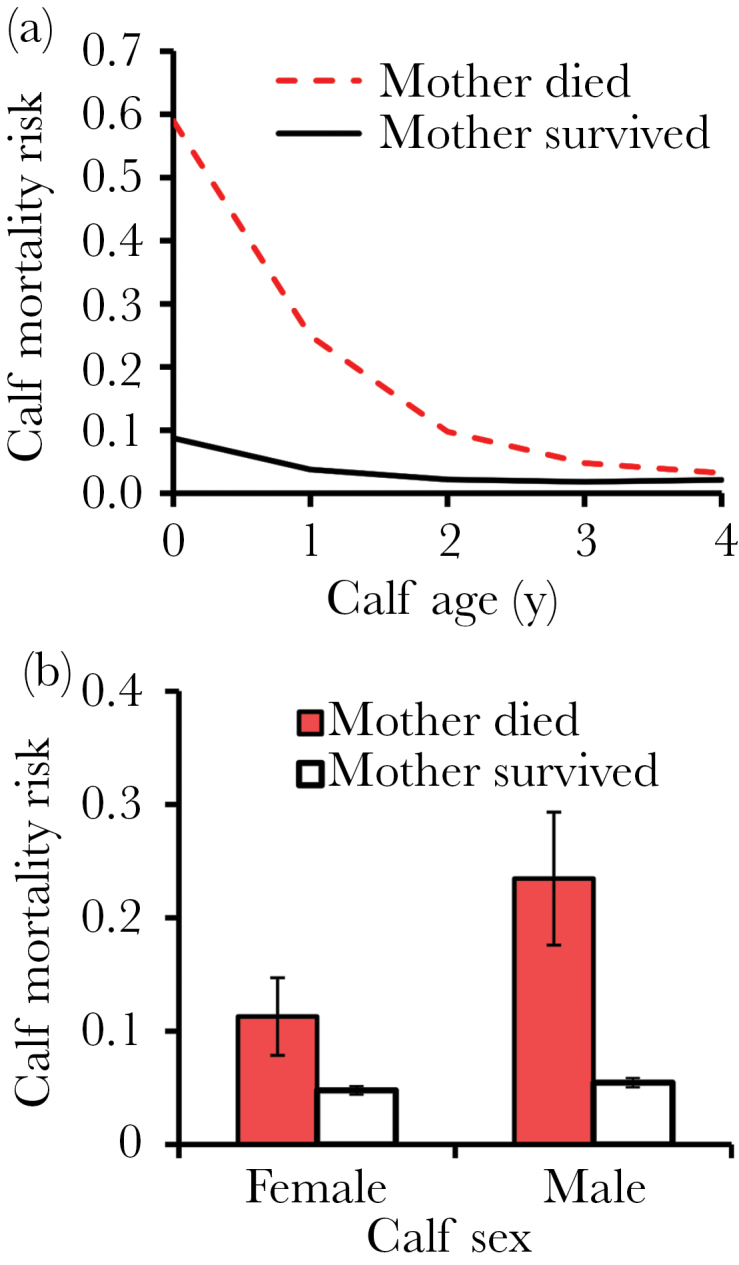
Consequences of mother loss for offspring risk of dying during dependency in Asian elephants. (a) Maternal death increased the calf’s probability of dying within the same or following calendar year (short-term effect) between birth and 5 years (*F*_1,6755_ = 35.59, *P* < 0.0001) and calf death probability decreased with increasing age (interaction between mother’s death and calf age: *F*_1,6755_ = 10.36, *P* = 0.0013). (b) Maternal death had a tendency of increasing male calf’s probability of dying more than female calf’s before age 5 (interaction between mother’s death and calf sex: *F*_1,6755_ = 2.25, *P* = 0.13). Sample size for the discrete time survival model is 7606 observations from 1813 calves and 838 mothers.

After controlling for all fixed and random terms, we found that the loss of a mother increased the immediate (during the same or following year) probability that a calf would die, but the strength of this effect varied depending on the age at which the offspring lost their mother (Table 2, Figure 2a). Maternal death increased calf mortality most in the same and the following year during the first 4 years of life, with the effect size decreasing steadily with calf age (odds ratios for calf death if the mother died, age 0: 10.42; age 1: 5.88; age 2: 3.31; age 3: 1.87; age 4: 1.05). There was also a nonsignificant tendency that the calf’s risk of dying after maternal death differed between the sexes (mother’s death × calf sex—interaction; Table 2). Although both male and female calves had increased risk of dying if their mother died early in their life, male calves had a 2.4 times higher mortality risk if their mother died before age 5 compared with female calves that lost their mother (*F*_1,6755_ = 2.25, *P* = 0.13, Figure 2b).

#### Delayed effects of mother loss

The delayed survival costs from maternal death (if the calf survived the same and the subsequent year) were smaller in magnitude and depended on the calf age. The analysis from age 3–5 years implies that the calf mortality risk was still increased at ages 3 and 4 even if at least 2 years had gone by after the mother’s death (mother’s death: *F*_1,3055_ = 5.03, *P* = 0.025; interaction: mother’s death × calf age: *F*_1,3055_ = 3.78, *P* = 0.052, Table 3) but not anymore at age 5. The odds ratios of calf death were high at ages 3 and 4, 7.21 and 2.63, respectively, but 0.96 at age 5.

**Table 3:** Discrete time survival model of the delayed effects of mother loss (mother died at least 2 years ago) on offspring risk of death during 3–5 (5.99) years in semicaptive timber elephants in Myanmar (total *n* = 3787 observations [1389 calves and 725 mothers])

Term	Estimate ± SE	Statistic (*F*_numdf,dendf_)	*P* value
Mother’s death	5.00±2.23	5.03_1,3055_	0.025
Calf age	7.04±1.32	23.99_1,3055_	<0.0001
Calf age^2^	−0.77±0.16	23.62_1,3055_	<0.0001
Birth cohort	Random variation	3.13_3,3055_	0.025
Mother’s death × calf age	−1.0075±0.52	3.78_1,3055_	0.052
Constant	−18.64±2.72		
Previous birth interval		1.75_3,3052_	0.15
Sex (female vs. male)	−0.21±0.15	1.86_1,3054_	0.17
Mother’s age	0.0062±0.0090	0.47_1,3054_	0.49
Mother’s age^2^	0.00065±0.00060	1.17_1,3053_	0.28
Birth order	−0.065±0.064	1.03_1,3054_	0.31
Mother’s origin (CB vs. WC)	0.11±0.17	0.44_1,3055_	0.51
Mother’s working area	Random variation	0.42_7,3054_	0.89

Estimates (positive reflect increasing mortality risk) are provided for variables and 2-level factors. Terms retained and rejected in the final model (determined using AIC) are shown above and below the constant, respectively. Mother’s identity was fitted as a random term. AIC, Akaike information criteria; SE, standard error.

Moreover, the calves that lost their mother during the most critical period (0–3 years), but survived beyond age 6 and were already tamed and their training had started, still showed some delayed negative effects of early mother loss on their current survival. These older juveniles and adolescents had higher risk of dying between ages 6 and 9 (odds ratio 5.08) than juveniles whose mothers survived until the calves were separated from their mothers and the calf reached age 4 (mother’s death: *F*_1,3311_ = 3.90, *P* = 0.048; interaction: mother’s death × calf age: *F*_1,3310_ = 1.21, *P* = 0.27; Table 4).

**Table 4:** Discrete time survival model of the delayed effects of mother loss (mother died between ages 0 and 3/was alive at least 4 first years) on offspring risk of death during 6–9 (9.99) years in semicaptive timber elephants in Myanmar (total *n* = 3950 observations [1085 calves and 637 mothers])

Term	Estimate ± SE	Statistic (*F*_numdf,dendf_)	*P* value
Mother’s death	1.63±0.82	3.90_1,3311_	0.048
Calf age	−0.30±0.15	3.99_1,3311_	0.046
Constant	−6.90±2.72		
Calf age^2^	0.25±0.17	2.27_1,3310_	0.13
Sex (female vs. male)	−0.49±0.39	1.55_1,3310_	0.21
Mother’s death × calf age	−1.21±1.10	1.21_1,3310_	0.27
Previous birth interval		0.84_3,3308_	0.47
Mother’s age	0.0083±0.025	0.11_1,3310_	0.74
Mother’s age^2^	0.0011±0.0019	0.36_1,3309_	0.55
Mother’s origin (CB vs. WC)	0.13±0.64	0.04_1,3311_	0.84
Birth order	0.027±0.16	0.03_1,3310_	0.86
Birth cohort	Random variation	0.17_3,3308_	0.92
Mother’s working area	Random variation	0.13_7,3311_	0.99

Estimates (positive reflect increasing mortality risk) are provided for variables and 2-level factors. Terms retained and rejected in the final model (determined using AIC) are shown above and below the constant, respectively. Mother’s identity was fitted as a random term. AIC, Akaike information criteria; SE, standard error.

## DISCUSSION

The Mother hypothesis proposes that animals that have the longest infant dependency and suffer from high costs of reproduction at old ages should have the longest postreproductive lifespans (Williams 1957). Specifically, such species may survive long after last birth because maternal death has significant negative fitness consequences for the survival of all dependent offspring, and any further reproductive attempts would thus not only risk own (maternal) survival but also that of all the already produced offspring that still depend on the mother for their survival. We investigated whether the long offspring dependency and higher costs of reproduction in older mothers could have selected for the relatively long postreproductive lifespan observed in Asian elephants (Lahdenperä et al. 2014). We found support for the long offspring dependency and high costs of maternal death for offspring survival but found no evidence for higher immediate costs of reproduction with maternal age per se measured as the age-specific risk of maternal death within 1 year after calving.

The Mother hypothesis was originally proposed to account for the evolution of menopause in humans (Williams 1957; Peccei 2001). A previous study of 2 preindustrial human populations found that the risk of dying from childbirth increases later in life, but the risk is low, such that women only have a 1–2% chance of dying from childbirth by age 50 and a projected estimate of 2–8% chance by age 70 (Lahdenperä et al. 2011). In humans, these risks, combined with the adverse effects of maternal loss on offspring survival, are estimated to be too weak alone to select for the evolution of menopause and the subsequent decades long postreproductive lifespan (Lahdenperä et al. 2011). Here, in this study, we found that female elephants do not show similarly increasing costs of reproduction with age as their probability of dying from calving-related causes within 1 year after giving birth was unaffected by maternal age per se. Thus, it seems that in semicaptive elephants, the costs of reproduction at old age are manifested through lower survival of calves born to older mothers (Table 2; Mar et al. 2012) and higher risks of stillbirths (Mar et al. 2012), but not through immediately decreased maternal survival after calving per se. Such lower survival and “quality” of offspring born to aged mothers alone are unlikely to select for the evolution of menopause as sometimes proposed in humans. However, despite that maternal mortality within a year following birth was not increased at old age, high reproductive investment at young ages (before the peak age of reproduction at 19) was found to subsequently accelerate general mortality rates of females at older ages (Hayward et al. 2014), and older females that suckled and raised a calf to weaning age at 5 also suffered from increased mortality risk within 5 years postbirth compared with females that did not reproduce at the same age (Robinson et al. 2012). Thus, reproduction in Asian elephants involves measurable costs to maternal mortality, but such costs do not simply manifest as immediately increased maternal mortality in older females as predicted by the Mother hypothesis.

Secondly, we found that offspring suffered both immediate and delayed adverse effects from their mother’s death, but the magnitude of these effects was related to the calf age. If the mother died when the calf was below age 5, the risk of calf death during the same or the subsequent year was multiplied. The risk of dying during the first living year was 10-fold, during the second year 6-fold, third year 3-fold, fourth year 2-fold, and finally at fifth year only 1.1-fold if the mother died during the same or the previous year. Such effects are of course expected given the dependency of newborn on maternal milk and protection, but the effects lasted beyond the adoption of solid food, which usually occurs gradually from around age 9 months onwards (Lee and Moss 2011). Besides such immediate effects on calf mortality, maternal death can have longer term consequences for the offspring if the lack of continued maternal care means lower body condition or health also for those offspring who did not die immediately after their mother. Such effects are well documented in some shorter-lived animals where maternal separation and lack of secure attachment are known to have long-term changes in brain, health, and behavior (e.g., Conti et al. 2012; Rivarola and Renard 2014) and humans where maternal loss can have long-lasting psychological, educational, and health consequences (e.g., Akerman and Statham 2011; Lahdenperä et al. 2011; Coneus et al. 2014). We also found delayed effects from maternal death: if the mother had died at least 2 years ago and the calf had survived the first years following maternal death, it nevertheless continued having an increased risk of mortality particularly at ages 3 and 4. Finally, as the most susceptible period for calf survival were the first 4 years of life, if the mother died during this time period and the calf survived until age 6, the calf survival was still reduced during adolescence, between ages 6 and 9 years. These results indicate that in our study population of Asian elephants the critical dependency on the mother lasted approximately 4 years, but the maternal loss also had some longer term consequences. This is in accordance with the average weaning age in Asian elephants (Mar 2007; de Silva et al 2013), the age when the calves make the transition to a diet of fully solid food (Mar 2007) and the average interbirth interval (Mar 2007; de Silva et al 2013) in elephants. In our study population also, the training begins and calves are separated from their mothers around ages of 4 and 5.

Our results are in line with findings reported for wild African elephants of Amboseli, Kenya, although the effects of mother loss in this species were even more detrimental than in our semicaptive population of Asian elephants with some human care available to the elephants, and no delayed effects of mother loss were investigated (Lee and Moss 2011). In Amboseli, if the mother died when the calf was below age 2, calves had no survival prospects and being orphaned between 2 and 9 years continued reducing the chances of survival (Lee and Moss 2011). Therefore, calves appeared to be dependent on their mothers for the first 5–10 years of life. Unfortunately, there are no comparable previous studies on Asian elephants, except for observations on wild elephant calves in Sri Lanka orphaned before the age of 4, which suggest that milk is important for normal growth at least up to this age despite consumption of herbaceous vegetation (de Silva et al. 2013).

We also found a trend that male calves are more vulnerable and have higher risk of dying after maternal loss than female calves. Although male and female calves seem to be relatively similar sized at birth in both Asian and African elephants, differences in growth rate between the sexes become obvious by age 2–5 years (Sukumar 2003c). Also, it has been observed that in African elephants there is a greater maternal investment in male than female calves during early years, as the males are more successful in their suckling attempts and have higher milk intake (Lee and Moss 1986). Thus, because of the higher growth rate in male calves, the early maternal loss may have more adverse effects on male than female calves.

The mechanisms accounting for the observed effects on offspring survival likely involve multiple pathways. Besides direct reductions in nutrition and protection, the effects of mother loss on calf mortality risk may include complex social effects. For example, there is evidence that orphaned calves become depressed (Poole et al. 2011) and calves that are separated from their mothers and other family members can have lasting trauma resulting in abnormal behavior (Slotow et al. 2000; Bradshaw et al. 2005). These social effects may be long lasting: individuals experiencing separation from family members and translocation during culling operations decades earlier performed poorly on systematic test of their social knowledge (Shannon et al. 2013). Consequently, delayed effects of early mother loss can be evident also in adulthood. Accordingly, it has been found among African elephants that slow growth during early lactation was associated with smaller adult size, later AFR, reduced lifetime survival and consequently limited reproductive output (Lee et al. 2013). Possibly males are also more likely to show stronger delayed effects in adulthood than females because size in adult male elephants is an important factor in mating success (Moss 1983; Poole 1989). Unfortunately, we were unable to study these long-term effects in adulthood because our sample size of calves that lost their mother during early years but nevertheless survived to adulthood was too low or these individuals were currently still too young to allow meaningful tests for measures of adulthood success. Of all calves that lost their mother before reaching age 4, and still survived to age 4 themselves, only 25% were known to have survived to age 20 to date (20 is the mean age at first female reproduction in the population; Lahdenperä et al. 2014). In contrast, of all calves that survived to age 4 alongside their mother, 52% were known to have survived to age 20 to date (many were censored before this age).

Moreover, we could not study whether mother loss after the first 5 years has negative short-term effects on calf survival. This is because the calves are separated from their mothers between ages 4 and 5 when the training begins. Some studies on other mammals show that maternal death after weaning can also have substantial negative effects on offspring survival. Although it is unclear if the methods were suitable for these kind of questions, a study of wild chimpanzees (*P. troglodytes*) in Tanzania shows that losing a mother decreased the likelihood of survival not only in infants but also in juveniles (5–13 years) and the effects were particularly strong among male offspring (Nakamura et al. 2014). Similarly, in red deer (*Cervus elaphus*), a mother’s presence affects offspring survival even after weaning (Andres et al. 2013), in females stronger than in males. However, although we could not investigate immediate effects beyond age 5, such effects—if existing—are likely to be of small magnitude given the observed strong decline in the effect size already during the first 5 years, such that we could not detect any statistically significant effects of mother death on calf immediate survival in the fifth living year and the odds ratio had shrank from 10-fold in the first year to 1.1 in the fifth year.

Our results imply that the Mother hypothesis could explain the first 5 years of postreproductive lifespan in Asian elephants, which is in accordance with the overall average postreproductive lifespan of 6 years in the entire population (in reproductive females dying at any age) (Lahdenperä et al. 2014). This period corresponds closely with the average interbirth interval in the population (Lahdenperä et al. 2014) and could therefore merely reflect adaptive birth spacing that ensures the success of the produced calf—which in the case of elephants is a long period. Interestingly, however, the postreproductive lifespan of females that survive up to old age in Myanmar timber elephants is considerably longer than that, at the population level 17 years after age of 47, when 95% of population fecundity has been realized and at the individual level 11 years for those females living beyond 40 years (Lahdenperä et al. 2014). This complicates the interpretation of the results in the light of the Mother Hypothesis. Packer et al. (1998) state that infant dependency at the age when the fertility drops in baboons and lions, 21 and 14 years, respectively, can explain the relatively short postreproductive lifespan of 5 and 1.8 years in these species, but there is no information on how long individuals of any age usually survive after last reproduction to provide a comparison to our study. In preindustrial humans, maternal death was also found to decrease offspring survival only during the first 2 years (Lahdenperä et al. 2011) and in killer whales during 2–3 years (Ward et al. 2009). Both species exhibit decades long postreproductive phases (Ward et al. 2009; Lahdenperä et al. 2014) and consequently the Mother hypothesis has been deemed unable to explain the entire postreproductive lifespans in these species.

So far, all evidence suggests that as in humans, female reproductive success in Asian elephants initially improves in their teens and reaches its peak already in early 20s, but in contrast to human women, it then declines gradually over the following decades (Hayward et al. 2014; Lahdenperä et al. 2014), although the postreproductive lifespan can be relatively long in females surviving to old age. Such declines in survival and annual fertility can be accelerated by high early-life reproductive investment (Hayward et al. 2014) or poor developmental conditions (Mumby HS et al., unpublished data) as has been also found in shorter-lived species (Gustafsson and Part 1990; Reid et al. 2003; Descamps et al. 2006; Nussey et al. 2006; Reed et al. 2008). Although the ability of mothers to produce robust offspring that survive to weaning age declines particularly fast in their 40s and 50s (Hayward et al. 2014) and general mortality rate of females accelerates, too (Figure 1a; Sukumar 2003b), we found no evidence that females would have benefitted from ceasing reproduction at these ages. This is because further female reproductive attempts did not entail an immediately increased risk of maternal mortality following birth that would have further jeopardized the survival chances of the previously produced offspring, and any future reproduce attempts at these ages therefore likely still contributed to a higher overall reproductive success. However, a possibility remains that although such late-life reproductive attempts did not increase the immediate mortality risk of the females, they might nevertheless have long-term health consequences. For example, high early-life reproductive rate appears to increase mortality risk decades later (Hayward et al. 2014).

This possibility is of interest in the light of another adaptive mechanism suggested to account for the evolution postreproductive longevity, proposed by the Grandmother hypothesis (Hawkes et al. 1998). If mothers can increase the success of their adult offspring by prolonging their care/help, then females with genes for living beyond the decline in fertility may produce more grandoffspring. Increasing evidence supports this idea in long-lived species with decades long postreproductive lifespans such as humans and killer whales (Lahdenperä et al. 2004; Foster et al. 2012). However, although in elephants, too, maternal presence has been linked to an increase in the daughter’s first calf’s survival (Moss and Lee 2011) and wisdom of old matriarchs have been found to be important for group survival and reproductive success (McComb et al. 2001; for killer whales see also Brent et al. 2015), specific quantitative data testing this hypothesis are still lacking. Such future analyses would be of interest to unravel whether the selection pressures for postreproductive survival differ between these species with differing timing of reproductive cessation and relative measures of postreproductive lifespan (such as PrR, Levitis and Lackey 2011) but similar social organization and high cognitive abilities or whether in elephants, too, it would “pay” for females to reduce reproductive rates at older ages in order to allow survival into more advanced ages.

## FUNDING

We thank the Leverhulme Trust (V.L., K.U.M.), ERC (V.L.), Academy of Finland (M.L.), NERC (K.U.M., V.L.), Rufford Foundation (K.U.M.), and Kone Foundation (M.L.) for funding our research.
